# The economic impact on families when a child is diagnosed with cancer

**DOI:** 10.3747/co.v15i4.260

**Published:** 2008-08

**Authors:** B. Miedema, J. Easley, P. Fortin, R. Hamilton, M. Mathews

**Affiliations:** * Dalhousie University, Family Medicine Teaching Unit, Fredericton, NB.; † Secteur des Sciences Humaines, Université de Moncton, Campus d’Edmundston, Edmundston, NB.; ‡ Division of Community Health and Humanities, Memorial University of Newfoundland, St. John’s, NL.

**Keywords:** Childhood cancer, economic effects, qualitative study, effects on work, travel expenses, out-of-pocket expenses

## Abstract

**Objective:**

In a study conducted in New Brunswick and Newfoundland and Labrador, we examined the economic impact on families caring for a child with cancer.

**Methods:**

We undertook semi-structured interviews with 28 French and English families with a child diagnosed with cancer in the last 10 years.

**Results:**

Families who care for a child with cancer incur considerable costs during the diagnostic, treatment, and follow-up care phases of the disease. Four major themes emerged from this qualitative study as contributing factors for these expenses: necessary travel; loss of income because of a reduction or termination of parental employment; out-of-pocket treatment expenses; and inability to draw on assistance programs to supplement or replace lost income. In addition, many of the decisions with regard to the primary caregiver were gendered. Typically, the mother is the one who terminated or reduced work hours, which affected the entire family’s financial well-being.

**Conclusions:**

For families with children diagnosed with cancer, financial issues emerged as a significant concern at a time when these families were already consumed with other challenges. This economic burden can have long-term effects on the financial security, quality of life, and future well-being of the entire family, including the siblings of the affected child, but in particular the mother. Financial assistance programs for families of seriously ill children need to be revisited and expanded.

## 1. INTRODUCTION

In Atlantic Canada, approximately 90 children between birth and 14 years of age are diagnosed with cancer annually 1. Although childhood cancer is rare, it is nevertheless the most common disease-related cause of death among children. Fortunately, the survival rate for children with cancer has increased dramatically since the late 1990s. Understandably, the psychological, sociologic, and financial effects of the disease can be extremely stressful for families 2. Few studies have been conducted to document these issues, particularly from the perspective of the families who care for a child with cancer.

## 2. BACKGROUND

The experience of pediatric cancer patients is different from that of adults with cancer, because the whole family—particularly the parents, and in some cases, the grandparents—are usually completely involved in the child’s illness 3,4. It has been reported that parents can develop posttraumatic stress disorder from dealing with a child’s illness 5,6. Siblings have reported feeling lost and ignored by parents who are preoccupied with the sick child and who may be absent from home for extended periods of time accompanying a child receiving treatment out of town. These feelings can lead to behavioural challenges in the siblings left at home 3.

In addition to the disruptions of family dynamics, families describe financial hardship associated with caring for a child with cancer. In one study, 37% of families reported that they were forced to borrow money to cover the extra cost of treatment related to the child’s illness 7. Another study reported that parents of children with cancer suffered greater financial hardship than did parents of children with other serious illness such as diabetes 8.

Barr and Sala 9 reported that few studies have specifically examined the out-of-pocket expenses incurred by families dealing with childhood cancer and other chronic diseases. A small qualitative Canadian study by Scott–Findlay and Chalmers 10 reported that, among other hardships, families with children who had cancer were required to travel 400 km on average (round trip) to receive treatment. Yantzi *et al.* 11 reported a relationship between the distance a family has to travel to the hospital for children with chronic illness and the quality of family relationships, because of the travel time and time spent away from home.

In the present study, we were interested in learning about the experiences of families in New Brunswick and Newfoundland and Labrador who had cared or were caring for a child with cancer, and the effect of this experience on the family’s financial well-being.

### 2.1 Study Setting

New Brunswick and Newfoundland and Labrador are unique in that, in both provinces, half the population lives in rural areas or small towns. As a result, many people must travel to receive specialized treatments. For example, in New Brunswick, pediatric cancer patients are usually treated out-of-province in either Nova Scotia or Quebec. In Newfoundland and Labrador, patients can attend the Janeway Hospital for Children in St. John’s, but the province is large, and travel from remote areas can involve journeys of hundreds of kilometres.

### 2.2 Health Care System

All Canadian residents are entitled to enrol in a provincial health plan (php). The php covers all medically necessary physician and hospital costs and the cost of drugs provided in hospital.

Prescription drugs provided outside a hospital setting are not covered by the phps. For cancer patients, these drugs may include oral chemotherapy agents that can be administered at home 12 or supportive drugs (such as antiemetics or pain medications) given to combat the side effects of treatment. Provincial drug insurance programs may offset the costs of some of these drugs for low-income families, and both New Brunswick and Newfoundland and Labrador have such programs 13. Out-of-pocket drug expenses may also be cost-shared through private supplemental health insurance programs (Blue Cross or Medavie, for example). Private supplemental medical insurance (including a prescription drug plan) is often offered as an employment benefit, and individuals can also purchase supplemental health insurance on their own (but usually at much higher premiums). Costs covered through private supplemental insurance policies vary and may require a 20%–30% co-payment 14. Overall, 20% of Canadians lack private supplemental health insurance, and the proportion is higher in the Atlantic Provinces 15. In 2001, it was reported that 30% of Newfoundland and Labrador residents and 32% of New Brunswick residents did not have private supplemental health insurance 16.

## 3. METHODS

A qualitative research method was chosen for this project. Using qualitative methods, researchers can study social and cultural phenomena in the context of people’s day-to-day lives and from the viewpoint of the participants. This approach permits researchers to address the uniqueness of the particular situation and to generate a hypothesis or theory 17.

### 3.1 Research Ethics Board Certification

The research protocol used in our study was reviewed and approved by the River Valley Health Research Ethics Committee and the research ethics boards of Memorial University of Newfoundland and Université de Moncton. All participants signed an informed consent form before commencing their interview.

### 3.2 Inclusion Criteria

For inclusion in the study, we recruited parents or caregivers whose children were 19 years or younger when diagnosed with cancer. The child’s diagnosis had to have occurred no more than 10 years before recruitment.

### 3.3 Recruitment Process

In New Brunswick, staff members at the Canadian Cancer Society–New Brunswick Division mailed a letter to the parents of children who had participated in summer camps for children with cancer. The parents, if interested, could directly contact the study team for more information and to set up an interview. Participants were also recruited through newspaper articles and other French and English media.

In Newfoundland and Labrador, participants were recruited with the assistance of Candlelighters Canada–Newfoundland and Labrador Division, a childhood cancer support foundation. A notice of invitation was posted in the Candlelighters newsletter, followed by a letter sent to 25 specific families that fit the inclusion criteria.

The recruitment phase was concluded when the research team agreed that demographic and linguistic diversity was achieved in the sample and that no new themes were emerging from the interviews. With a total response rate to the individual mailings of approximately 30%, 9 anglophone and 12 francophone parents in New Brunswick and 7 anglophone parents in Newfoundland and Labrador participated in the study. Participants included one or both parents or caregivers.

### 3.4 The Interview

A semi-structured format was used to guide the interviews with the participants. The interview questions were all open-ended. The interview schedule started with general questions and then moved to questions about social supports, the effect of cancer on the child and the family, and specific questions about the economic effects of cancer. Most interviews took place in the participants’ homes, although a few took place in one of the research offices. In general, the interviews lasted between 1 and 2 hours. All participants agreed to have their interviews audiotaped on a digital recorder. After the interview was completed, the participants were asked to complete a sociodemographic form.

### 3.5 Data Analysis

All interviews were transcribed verbatim, and a rigorous constant comparative thematic analysis was applied 18. In a thematic analysis, researchers identify themes and common patterns among the experiences of the participants 19,20.

The three researchers and their three assistants all read 6 selected transcripts representing the three distinct interview groups: English New Brunswick, French New Brunswick (translated transcripts), and English Newfoundland and Labrador. All six researchers coded the transcripts independently. The researchers then convened for a 2-day team meeting to discuss the codes. Most codes were easily agreed on; in cases where disagreement arose, team discussions ensued to reach a consensus about the code. An English coding scheme was developed, and this coding scheme was used to analyze the rest of the transcripts from all three interview groups. The coding scheme was slightly revised and updated during two subsequent teleconferences.

Of the French interviews, 8 were translated into English by an official translation agency. Four interviews were coded in French by a bilingual researcher using the English coding scheme.

### 3.6 Confidentiality

Because of the relatively small number of pediatric cancer cases and the small population in the two provinces, as little detail as possible regarding the identities of the study participants is revealed here. Quotes may have been slightly modified to ensure that no identifying information is disclosed.

## 4. RESULTS

### 4.1 Profile of the Study Population

Of the 28 families, 5 had children still in active treatment at the time of the interview, 3 had experienced the death of their child, and the rest had children in the follow-up care stage of the cancer care continuum. Just over half (57%) of the families lived in rural areas, and almost three quarters considered themselves to be religious (68%) at the time of the interview. Most of the parents were married, with an average of 2 children, and they had postsecondary educations and good incomes (see Table I).

Among the parents, 17 mothers (61%) were working full- or part-time and 11 (39%) were not working (1 retired, 7 not in the labour force, 3 on sick leave or stress leave) at the time of the interview; 24 fathers (86%) were working full- or part-time, 2 (7%) were seasonal workers, and 2 (7%) were not in the workforce.

The age of the affected children at the time of diagnosis in New Brunswick ranged from 6 months to 17 years. In Newfoundland and Labrador, the age of the affected children at diagnosis ranged from 3 to 16 years. The most common diagnosis was acute lymphocytic leukemia. All children had received chemotherapy, a few had received radiation therapy, and a few had undergone bone marrow transplantation.

### 4.2 Themes Related to Economic Effects

The four major themes that emerged as contributing factors to the severe economic effects on the families were

- travel expenses for treatment and follow-up care,
- loss of income because of a reduction or termination of parental employment,
- out-of-pocket expenses for treatment, and
- an inability to draw on programs for assistance or income supplementation.

#### 4.2.1 Travel Costs

Of the 28 families interviewed, all but 3 were required to travel to other cities, frequently out-of-province, for treatments. In Newfoundland and Labrador, all families traveled to the Janeway Children’s Hospital in St. John’s. Most of the New Brunswick families traveled to the iwk Health Centre in Halifax, Nova Scotia. Some families living in western and northern New Brunswick traveled to children’s hospitals in Quebec. A few parents whose children underwent very specialized treatments such as bone marrow transplantation, traveled to Ontario hospitals.

It was not uncommon for parents to report having to travel immediately after a medical consultation to a large children’s hospital without returning home for weeks or sometimes months. Parents were then forced to make alternative arrangements for other children at home and (if they were working for pay) with their employers. As one parent said, “We just had to jump in the van and drive to [city] with literally the clothes on our backs and a few dollars. It was all we had. When we got there, we did not think about where we [were] going to stay” (interview 7).

The costs associated with travel and accommodations were substantial for many parents. One father said, “Financially, it set us back 10 years because of the loss of my salary and the wretched trips” (interview 9). Many families were able to take advantage of reduced-rate accommodations such as the Ronald McDonald House or hospital rates at local hotels; however, because of prolonged stays, the costs still added up for these families. As one mother described it, “even though you had Ronald MacDonald house to stay at for $11 a night, of course the phone bill[....] Every day, every report, we phoned home. Parking, meals at the hospital, and we tried to get groceries and eat when we could at Ronald MacDonald house, but that was ... you know, if I was going to take a guess, I would say it was couple thousand dollars for those three weeks” (interview 14).

#### 4.2.2 Work-Related Issues

**FIGURE 1:**
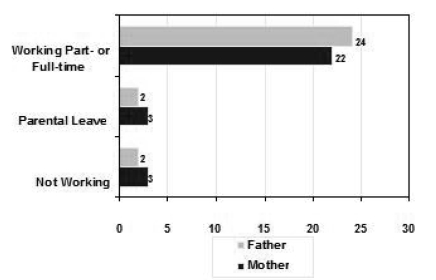
Employment of parents of children with cancer at the time of diagnosis.

**FIGURE 2:**
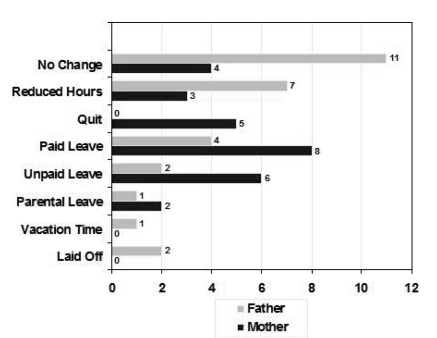
Employment status change of parents of children with cancer during the treatment phase.

In this study, caring for a child with cancer greatly affected the work patterns of the parents in general, but particularly the work patterns of the mothers. The work status of the parents at the time of their child’s cancer diagnosis and during treatment shows a considerable gendered change (see Figures 1 and 2). At the time of diagnosis, 24 fathers and 22 mothers were working for pay either full-time or part-time. Of the fathers, 61% reported that their work hours changed during the child’s treatment; among the mothers, the proportion was 86%.  Five mothers (18%) simply stopped working altogether.

One father described why his wife stopped working and looked after their sick child: “[She] is a nurse, and our salaries are pretty much equal. She earns a little bit more than I do[....] If I left for a year, six months or two years, I would have to transfer a lot of the projects to somebody else. It would be hard for me to start again, but it seems to be a lot easier for her [...] plus, she’s the mother.” During treatment, this mother was unable to continue to work. The father said, “[She] tried to work through [the child’s treatment] for a few months, and then she had to go on sick leave[....] The company denied her benefits. They said, ‘You’re not sick, your son is.’” (interview 13).

Self-employed parents often experienced an immediate loss of income: “There were times, you know, when we weren’t able to work the hours that we normally work, so there was much less money coming in[....] If you do not go to work, you don’t get paid” (interview 2).

#### 4.2.3 Out-of-Pocket Medical Costs

Not only do travel, lodging, and meals away from home add to costs, but so too do medications and medical supplies not covered by the php. Private supplemental health insurances covers some medication and supply costs, but parents without insurance must pay for out-of-hospital drugs and medical supplies themselves. Many of the interviewees spoke of spending many thousands of dollars for treatment equipment such as feeding tubes, needles, and medication. One mother was so overwhelmed by these costs for medical supplies for her child that she felt “the last thread snapped” when she was negotiating with the supplemental private health insurer on what they would cover and what they would not cover. This mother was ultimately forced to cancel her daughter’s Registered Education Savings Plan and to withdraw the funds to pay for medical expenses.

#### 4.2.4 Inability to Draw On Income Support Programs

Few parents were able to draw on formal programs for financial support. Only 1 parent remarked that supplemental private health insurance provided a per diem when the child was hospitalized; in most cases, however, the parents were unable to find any respite from the demands on their finances. Many parents reported that programs were contingent on the parent being able to “look for work” and that financial assistance was based on “previous earnings.”

One mother described her struggle with the federal Employment Insurance program: “We [husband and wife] tried to get on unemployment, but we couldn’t get that. We tried to get social assistance to help us, and they did a little bit, but it wouldn’t be enough to butter your bread, because they had said something about ‘You made too much money the month before.’” She noted that her husband had been laid off just before their daughter’s diagnosis of cancer: “They gave us the weekend off to pack up and to drive to the iwk. We went to the unemployment office to see if they would help us because he was laid off, but they refused. We did not lie about anything. I told him that my daughter had just found out that she has cancer, and we are leaving for Halifax. But they said there is nothing we can do[....] So you go with the credit cards you have” (interview 3).

## 5. DISCUSSION AND CONCLUSIONS

Our research clearly demonstrates the severity of the negative financial effects on families in New Brunswick and Newfoundland and Labrador when they care for a child with cancer. The emerging theory from this research is that government programs are inadequate to support families who care for children with catastrophic illnesses such as cancer. Although none of the interviewed families suggested that they had to withdraw care from their child because of financial constraints, we demonstrated that many parents struggled with financial hardship and that these concerns imposed additional stresses on the families.

Debts accrued over the course of the treatment, and the follow-up phase of the disease could have long-term effects on the financial stability of the family. Many families discussed how they were still paying off debts years after the treatment. A few parents even discussed having to re-mortgage their homes or to take money out of registered retirement or education savings plans to pay for the medical and out-of-pocket expenses.

Care for a child with cancer was, not surprisingly, gendered. It was most often the mother who reduced or terminated employment to care for the sick child, regardless of prior earning power in the family. Her reduced income not only was responsible for the immediate drop in family income, but also potentially jeopardized her future and retirement earnings. Inability to contribute to a registered retirement savings plan or a company pension plan could also affect her income in old age 21.

Caregivers in the formal health care system need to be aware that parents of pediatric cancer patients not only have to deal with the stress of the illness, but also with stressors in their immediate environment, which can be very severe 6,11.

Canada lacks support programs for parents caring for a child with a catastrophic illness. The federal government has introduced a “compassionate leave” program, but that program is geared toward caring for a spouse or parent with a terminal illness 22. Parents who care for a child with cancer are particularly vulnerable to financial ruin at the time of cancer treatments and, in most cases, for many years afterward, because cancer survivorship for children increases with technological and medical advances. Canada needs to develop programs for parents who care for children with catastrophic illnesses so that parents do not have to struggle financially to properly care for the child with cancer and that child’s siblings.

**TABLE I:** Income and education of parents of children with cancer at time of interview

	NB	NL	*Total [*n *(%)]*
Education
Mother
High school or less	2	2	4 (14)
Professional diploma	4	3	7 (25)
Undergraduate degree	6	1	7 (25)
Graduate/professional degree	8	1	9 (32)
Did not wish to answer	1	0	1 (04)
Father
High school or less	6	3	9 (32)
Professional diploma	3	3	6 (21)
Undergraduate degree	6	1	7 (25)
Graduate/professional degree	5	0	5 (18)
Did not wish to answer	1	0	1 (04)
Annual family income			
$20,000 or less	0	1	1 (04)
$20,001 to $30,000	4	0	4 (15)
$30,001 to $40,000	0	0	0
$40,001 to $50,000	3	1	4 (15)
$50,001 to $60,000	2	1	3 (11)
$60,001 to $70,000	1	1	2 (08)
$70,001 to $80,000	2	0	2 (08)
$80,001 or more	7	2	9 (32)
Did not wish to answer	2	1	3 (11)

NB = New Brunswick; NL = Newfoundland and Labrador.
